# Stretch-Induced Hypertrophy Activates NFkB-Mediated VEGF Secretion in Adult Cardiomyocytes

**DOI:** 10.1371/journal.pone.0029055

**Published:** 2011-12-13

**Authors:** Anna Leychenko, Eugene Konorev, Mayumi Jijiwa, Michelle L. Matter

**Affiliations:** 1 Department of Cell and Molecular Biology and Center for Cardiovascular Research, John A. Burns School of Medicine, University of Hawaii, Honolulu, Hawaii, United States of America; 2 Department of Molecular Bioscience and Bioengineering, University of Hawaii at Manoa, Honolulu, Hawaii, United States of America; 3 Pharmaceutical Sciences, University of Hawaii-Hilo College of Pharmacy, Hilo, Hawaii, United States of America; University of Frankfurt - Hospital Frankfurt, Germay

## Abstract

Hypertension and myocardial infarction are associated with the onset of hypertrophy. Hypertrophy is a compensatory response mechanism to increases in mechanical load due to pressure or volume overload. It is characterized by extracellular matrix remodeling and hypertrophic growth of adult cardiomyocytes. Production of Vascular Endothelial Growth Factor (VEGF), which acts as an angiogenic factor and a modulator of cardiomyocyte function, is regulated by mechanical stretch. Mechanical stretch promotes VEGF secretion in neonatal cardiomyocytes. Whether this effect is retained in adult cells and the molecular mechanism mediating stretch-induced VEGF secretion has not been elucidated. Our objective was to investigate whether cyclic mechanical stretch induces VEGF secretion in adult cardiomyocytes and to identify the molecular mechanism mediating VEGF secretion in these cells. Isolated primary adult rat cardiomyocytes (ARCMs) were subjected to cyclic mechanical stretch at an extension level of 10% at 30 cycles/min that induces hypertrophic responses. Cyclic mechanical stretch induced a 3-fold increase in VEGF secretion in ARCMs compared to non-stretch controls. This increase in stretch-induced VEGF secretion correlated with NFkB activation. Cyclic mechanical stretch-mediated VEGF secretion was blocked by an NFkB peptide inhibitor and expression of a dominant negative mutant IkBα, but not by inhibitors of the MAPK/ERK1/2 or PI3K pathways. Chromatin immunoprecipitation assays demonstrated an interaction of NFkB with the VEGF promoter in stretched primary cardiomyocytes. Moreover, VEGF secretion is increased in the stretched myocardium during pressure overload-induced hypertrophy. These findings are the first to demonstrate that NFkB activation plays a role in mediating VEGF secretion upon cyclic mechanical stretch in adult cardiomyocytes. Signaling by NFkB initiated in response to cyclic mechanical stretch may therefore coordinate the hypertrophic response in adult cardiomyocytes. Elucidation of this novel mechanism may provide a target for developing future pharmacotherapy to treat hypertension and heart disease.

## Introduction

Cardiovascular diseases such as myocardial infarction and hypertension often present with the development of cardiac hypertrophy. Hypertrophy is characterized by extracellular matrix (ECM) remodeling and enhanced growth of adult cardiomyocytes [1]. Increased mechanical stretch or volume overload promotes adult cardiomyocyte hypertrophy [2]. *In vivo* mechanical stretch induces growth and remodeling within the hemodynamically overloaded myocardium [3], [4]. This can be partially modeled *in vitro* when cardiomyocytes are cultured on ECM-coated flexible membranes and subjected to mechanical stretch that is similar to stretch overload *in vivo* [5]. In these assays, mechanical stretch of cardiomyocytes activates several hypertrophic responses including increased gene expression of brain natriuretic peptide [BNP] and atrial natriuretic peptide [6], endothelin-1 [7] and upregulation of growth factors and cytokines [8], [9].

In the heart, Vascular Endothelial Growth Factor (VEGF) is regulated by environmental stresses such as hypoxia [10] and mechanical stress [11], [12]. Recent studies on VEGF signaling suggest that while this cytokine promotes angiogenesis, it is also a primary regulator of cardiomyocyte function [13]. For example, VEGF levels are elevated in the sera of patients with acute myocardial infarctions [14] and VEGF preserves cardiac function post-infarction [13]. In addition, intramyocardial VEGF expression provides a significant improvement in cardiac function after permanent coronary artery occlusion via upregulating the genes that drive the compensatory hypertrophic response [15].

The mechanism of how hypertrophy activates VEGF secretion in adult cardiomyocytes *in vivo* or *in vitro* is unknown. *In vitro* mechanical stretch promotes VEGF secretion in neonatal cardiomyocytes; however the molecular mechanism responsible for VEGF secretion has not been investigated in these cells [11], [16]. Moreover, whether stretch activates VEGF secretion in the adult cell and the molecular mechanism mediating stretch-induced VEGF secretion in either neonatal or adult cardiomyocytes has not been elucidated. Adult cells retain their phenotype and are less likely to de-differentiate compared to neonatal cells. In addition, isolated adult cells do not demonstrate general increases in contractile proteins or ANP that occur in the neonatal phenotype [17], [18]. Thus, hypertrophic signaling in adult cells is more likely to represent the *in vivo* state. Therefore, we chose adult primary cardiomyoctes to investigate whether mechanical stretch activated VEGF secretion and to elucidate the molecular mechanism. We demonstrate here that cyclic mechanical stretch-induces VEGF secretion via the NFkB signaling pathway in adult cardiomyocytes.

## Materials and Methods

### Animals

Experiments were performed on Sprague-Dawley rats (6-8 weeks old, male). Animals were obtained from The Jackson Laboratory (Bar Harbor, ME). All animals received care in compliance with the principles of laboratory animal care and use formulated by the Institutional Animal Care & Use Committee.

### Antibodies and Inhibitors

Antibodies directed against phospho-p44/42 MAPK (ERK1/2) [T^202^/Y^204^ and T^185^/Y^187^] XP (#4370), p44/42 MAPK (ERK1/2) (#4695), phospho-AKT [T^308^] (#9275), AKT (#9272), phospho-IkBα [S^32^/S^36^] (#9246), or IkBα (#9242) were purchased from Cell Signaling (Danvers, MA). Antibodies directed against NFkB p65 (#372) or β-actin (#1516) were purchased from Santa Cruz Biotechnology (Santa Cruz, CA). Mouse monoclonal anti-TATA-binding protein (TBP) (#51841) was purchased from Abcam (Cambridge, MA). HRP-Goat anti-rabbit IgG (H+L) Conjugate (#62-1820) and HRP-Goat anti-mouse IgG (H+L) (#62-6520) were purchased from Invitrogen (Carlsbad, CA). Secondary antibodies against rabbit (#926-32213) and mouse (#926-32212) were obtained from LI-COR Biosciences (Lincoln, NE). All antibodies were diluted in 3%BSA/TBS (0.1% Tween 20) buffer at 1∶1000 for the primary, and 1∶10000 for the secondary.

Cell-permeable peptide-inhibitor of the translocation of the NFkB active complex into the nucleus, SN50 (P-600), and its inactive peptide-control, SN50M (P-601) were purchased from ENZO LS (Farmingdale, NY). SN50 and SN50M were dissolved in media at 15 µg/ml and 30 µg/ml. A selective inhibitor of the mitogen-activated protein kinase kinases (MAPKK), MEK-1 and MEK-2, U0126 (U-6770), and a specific inhibitor of phosphatidylinositol 3-kinase (PI3K), LY294002 (L-7962), were purchased from LC Labs (Woburn, MA). U0126 and LY294002 were dissolved in DMSO.

### Cardiomyocyte Isolation and Culture

Primary culture of cardiac myocytes was prepared from the whole adult rat hearts. The procedure was modified from Piper *et al* [19] and optimized to obtain a high yield of viable ARCMs. Briefly, rats were anesthetized with isofluorane and injected with 200 U heparin (5.4 µg/µl). Hearts were then excised, cannulated, and perfused for 30 min with perfusion buffer [pH 7.3; containing HEPES (25 mM), NaCl (110 mM), KCl (2.6 mM), creatine (5 mM), taurine (20 mM), MgSO_4_ (1.2 mM), KH_2_PO_4_ (1.2 mM) and glucose (11 mM)] supplemented with collagenase Type II (6 mg/ml; #17101-015, Invitrogen, Carlsbad, CA). Perfused hearts were minced in DNase I solution (50 µg/ml #D4513, Sigma, St. Loius, MO) supplemented with 1% BSA, strained and layered over a 4% BSA gradient. Sedimented ARCMs were resuspended in culture media, Medium 199 (#11150, Invitrogen, Carlsbad, CA) supplemented with HEPES (25 mM), BSA (0.2%), human insulin (0.6 mg/ml), PenStrep (1%), taurine, creatine and carnitine. Cells were plated on 6-well culture plates pre-coated (overnight, 4°C) with 15 µg/ml natural mouse laminin (#23017-015, Invitrogen, Carlsbad, CA), and incubated at 37°C, 5% CO_2_. After 1 h of plating, media was replaced and cells were incubated for 24 h before the use in the study.

### MTT Assay

Viability of cardiomyocytes in culture was assessed using the MTT assay [20], [21]. The assay measures the ability of an active mitochondrial enzyme to reduce the MTT substrate (yellow to blue) in live cells. Isolated primary cardiomyocytes were plated in serum-free conditions on 48-well plates pre-coated with laminin. After 24 h or 48 h of culture, 0.5 mg/ml MTT substrate (Thiazolyl Blue Tertrazolium Bromide) was added and cells were incubated for additional 4 h, and then solubilized with 10% SDS/HCl (0.01N) overnight. Absorbance was measured at 595 nm.

### Cyclic Mechanical Stretch

Flexible-bottomed tissue culture plates (UF-4001U) were purchased from FlexCell International (Hillsborough, NC). After a media change, pulsatile stretch of ARCMs was performed in a Flexcell FX-4000 (V4.0) strain unit at an extension level of 10% at 30 cycles/min for 24 h and 48 h. This procedure has previously been reported to mimic hypertrophic stress *in vitro* [9]. During the duration of stretch, cells were kept in the incubator at 37°C, 5% CO_2_. Control (non-stretched) ARCMs were cultured identically without the stretch. At the conclusion of the experiment, samples of conditioned culture media were collected for measuring VEGF levels via ELISA and cell lysates were collected for immunoblotting.

### Subcellular Fractionation: Cytoplasmic and Nuclear Fractions

A stepwise extraction of four distinct subcellular proteomes from one sample based on their solubility was performed using ProteoExtract™ Subcellular Proteome Extraction Kit (#539790, EMD Chemicals, Gibbstown, NJ). Briefly, stretched and non-stretched ARCM attached to laminin-coated plates were sequentially incubated with four extraction buffers, and fractions containing cytosolic, membrane, nucleic or cytoskeletal proteins were collected at each step.

Immunoblotting analysis was performed on cytosolic and nucleic fractions as described below. Equal volume (20–25 µl) of each fraction was used for analysis. Tata-binding-protein (TBP) was used as a nuclear fraction marker [22].

### Immunoblotting

Immunoblotting analysis was performed as previously described [23]. Briefly, cell lysates of stretched and non-stretched cardiomyocytes cultured with or without inhibitors were centrifuged at 14,000 rpm for 15 min. Supernatants containing equal amounts of total soluble proteins were separated by SDS-PAGE and transferred to nitrocellulose membranes for immunoblotting with specific antibodies. Band intensities were quantified by scanning densitometry.

### Adenovirus transduction of Dominant Negative Mutant IKBα

Twenty-four hours after isolation ARCMs were transduced with an IkBα dominant negative mutant (IkBα -S32A/S36A; DNM IkBα) adenovirus (Vector Biolabs, Philadelphia, PA) at 10^8^ PFU/ml or control. Stretch experiments were performed 24 h later. Culture media was changed and cells were stretched or non-stretched for an additional 24 h. Media was collected and analyzed via ELISA for secreted VEGF or BNP levels as described below. Whole cell lysate or cytoplasmic and nuclear fractions were extracted and analyzed via immunoblotting assay for total IkBα or for p65-NFkB activity as described above.

### Quantification of VEGF or BNP levels in cultured media by ELISA

Samples of media from stretched and non-stretched primary cardiomyocytes were collected (1 ml from each well), and centrifuged at 14,000 rpm for 15 min to pellet off dead cells and debris. The supernatants were analyzed for the levels of secreted VEGF protein via Quantikine mouse/rat VEGF ELISA kit (#MMV00, R&D Systems, Minneapolis, MN) or BNP protein via AssayMax Rat BNP-32 (rBNP-32) ELISA kit (#ERB1201-1, AssayPro, Saint Charles, MO) according to the manufacturer instructions.

The VEGF ELISA kit recognizes both VEGF A and VEGF B forms.

### Chromatin Immunoprecipitation (ChIP) Assay

ChIP assays were performed by using the EZ-ChIP Assay Kit (Upstate Biotechnologies, Millipore Co. Billerica, MA) following the manufacturer's instructions. Briefly, ARCMs were cultured for 24 h and then either stretched or non-stretched for additional 24 h. Next 37% formaldehyde was added directly to the media of above cultured ARCMs to a final concentration of 1% RT for 10 min to crosslink the proteins to the DNA. Cross-linking was then stopped by adding 2.5 M glycine to a final concentration of 0.125 M, washed twice in ice cold PBS, scraped, centrifuged to pellet and re-suspended in SDS lysis buffer containing Protease Inhibitor Cocktail II. Native chromatin was sonicated to shear cross-linked DNA to ∼200–1000 base pairs in length. Sheared cross-linked chromatin then was pre-cleared with Protein G Agarose by incubating for 1 h at 4°C with rotation. After pelleting agarose by centrifugation, 1% volume of each sample was removed as “input fraction” (non-immunoprecipitated) and remaining supernatant was immunoprecipitated (IP) with antibodies against NFkB p65 or control antibodies overnight at 4°C with rotation. 60 µl of Protein G Agarose was added to recover protein/DNA complexes at 4°C for 1 h. Beads were pelleted and washed sequentially in the low salt buffer, high salt buffer, LiCl wash buffer and Tris-EDTA buffer. Following washing of the beads, immunoprecipitated protein/DNA complexes were analyzed via immunoblot analysis with p65 NFkB. Reversal of protein/DNA crosslinks was carried out by heating samples at 65°C overnight. The elution was then digested with RNAase at 37°C for 30 min and with proteinase K at 45°C for 2 h. DNA was purified with provided Spin Filters according to the kit instructions. Real Quantitative PCR (RT-qPCR) was performed with 10 µl of immunoprecipitated DNA using the following conditions: 1cycle of 95°C for 2 min, 30cycles of 95°C for 30 sec, 68°C for 5 sec. The primers (designed by Genomics Shared Resource; University of Hawaii Cancer Center) used were rat VEGF promoter +420 bp: 5′-CGACTGGTCCGATGAAAGAT-3′ Forward (F) and 5′-CGAACAGAGAGAGGGACAGG-3′ Reverse (R).

### Pressure Overload Studies

8-week old males were subjected to ascending aortic constriction or the sham operation. This model causes LV hypertension and hypertrophy. Hearts were isolated at 10 days post-surgery, perfused and fixed in 4& paraformaldehyde (PFA), dehydrated, and embedded in paraffin. Immunostaining with an anti-VEGF antibody (Santa Cruz Biotechnologies, Santa Cruz, CA) was performed using the Vectastain DAB kit protocol (Vector Laboratories, Burlingame, CA).

### Statistical Analysis

ELISA and densitometric readings were subjected to statistical analysis. Differences between the mean values and the densitometric readings were analyzed by ANOVA followed by Bonferroni's test for multiple comparisons between pairs. Values of P<0.05 indicated statistical significance. P-values are abbreviations as follows, ***P<0.001, **P<0.01, *P<0.05, or non-significant (n.s.). At least three independent experiments were performed for each data set and combined for statistical analysis.

## Results

### Cyclic mechanical stretch promotes VEGF secretion in adult cardiomyocytes

**Figure 1:**
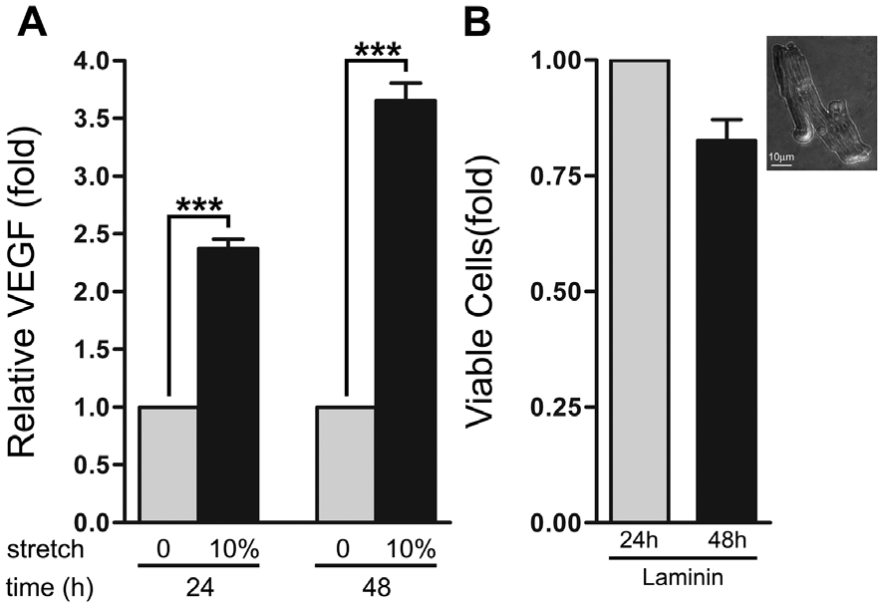
Cyclic mechanical stretch induces VEGF secretion in primary ARCMs. **A**) Cyclic mechanical stretch for either 24 h or 48 h induces a significant increase in VEGF secretion in primary ARCMs attached to laminin compared to non-stretched controls. Stretched (10% stretch) and non-stretched control (0% stretch) cells were allowed to adhere to laminin for 24 h prior to initiating experiments. VEGF concentration in the conditioned media of non-stretched and stretched ARCMs was analyzed by ELISA. ***P<0.001; One-Way ANOVA with a Bonferroni post-test was used to determine the statistical significance of data. The values represent the average of three independent experiments. **B**) Isolated ARCMs remain viable in culture when attached to laminin. Cell cultures were examined for their ability to reduce MTT after 24 h or 48 h in culture. Fold induction in relative mitochondrial activity represents the amount of viable cells at each time point. ***P<0.001; One-Way ANOVA with a Bonferroni post-test was used to determine the statistical significance of data. **Insert:** The isolation procedure yielded a >90% pure ARCM population. Phase contrast image of isolated, primary ARCMs 24 h after binding to laminin demonstrate that ARCM differentiated morphology is maintained as indicated by the rod-shaped, branched striated cell examined at 40X magnification.

*In vivo* cardiomyocytes secrete VEGF upon hypoxia and mechanical stretch [24]. To investigate whether cyclic mechanical stretch induced VEGF secretion *in vitro*, we measured the concentration of VEGF in the conditioned media from isolated adult rat cardiomyocytes (ARCMs) subjected to cyclic stretch or non-stretched controls. ARCMs attached to laminin-coated flexible-bottomed tissue culture plates were subjected to pulsatile stretch using a Flexcell FX-4000 (V4.0) strain unit at an extension level of 10% at 30 cycles/min that was optimized to promote hypertrophic responses [8], [9], [25]. Cyclic stretch significantly increased the concentration of VEGF in the conditioned media by approximately 2.2-fold at 24 h and 3-fold at 48 h compared to their respective non-stretch controls **(** **Figure 1A** **)**.

To determine if primary ARCMs bound to laminin survive over time in culture, we assessed cell survival by the MTT assay, which measures cell viability by detecting the ability of a mitochondrial enzyme to reduce its substrate as demonstrated by a colorimetric reaction [20]. ARCMs attached to laminin survive in culture for at least 3 days (**Figure 1B**). ARCMs were obtained at >90% purity and retained their differentiated elongated rod-shaped branched morphology with distinct striations when plated on a laminin matrix (**Figure 1** **insert**).

### Cyclic mechanical stretch activates the NFkB, MAPK/ERK1/2 and PI3K pathways in ARCMs

**Figure 2:**
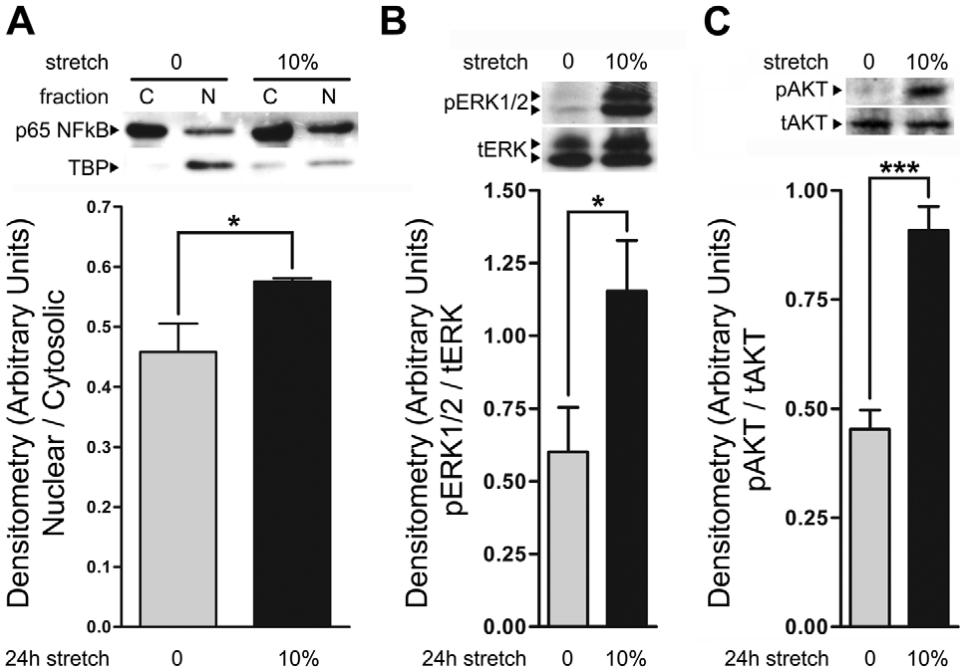
Cyclic mechanical stretch activates the NFkB, MAPK/ERK1/2 and PI3K pathways in ARCMs. **A**) Isolated ARCMs attached to laminin were subjected to 24 h of cyclic mechanical stretch (10% stretch). Non-stretched control (0% stretch) ARCMs attached to laminin were incubated under identical conditions. Cells were then lysed, fractionated into cytosolic (C) and nuclear (N) fractions and immunoblotted with an anti-NFkB p65 antibody. In stretched ARCMs, the nuclear fraction contained significantly more of the NFkB p65 subunit compared to the non-stretched controls. Relative intensity of nuclear to cytoplasmic (N/C) fraction was determined. An antibody to the anti-TATA-binding protein (TBP) was used to determine fractionation efficiency. Cyclic mechanical stretch for 24 h induced a significant increase in MAPK/ERK1/2 and PI3K activity in isolated ARCMs attached to laminin. Isolated ARCMs were allowed to attach to laminin for 24 h and subsequently subjected to 24 h of cyclic mechanical stretch (10% stretch) or no stretch (0% stretch). Cells were lysed and immunoblotted for (**B**) ERK1/2 activity using an antibody against phospho-ERK1/2 (pERK) or a total ERK1/2 antibody (tERK) or for (**C**) PI3K activity via an antibody against phospho-AKT (pAKT) or total AKT (tAKT). Relative intensity of pERK/tERK or pAKT/tAKT was determined. *P<0.05; ***P<0.001; One-Way ANOVA with a Bonferroni post-test was used to determine the statistical significance of data. The values represent the average of three independent experiments.

Integrin attachment to the ECM can activate NFkB, MAPK/ERK1/2, and PI3K pathways [26]. Upon NFkB activation, the NFkB heterodimer complex including the p65-subunit is translocated to the nucleus where it upregulates a number of genes that affect cell survival and cardiac hypertrophy. We next examined whether *in vitro* hypertrophic cyclic mechanical stretch of adult cardiomyocytes may directly influence NFkB subcellular distribution and activity. Cytosolic and nuclear fractions from non-stretched and stretched ARCMs were blotted with an anti-NFkB p65 antibody. As shown in stretched ARCMs, more NFkB p65 was present in the nuclear fraction compared to non-stretched controls (**Figure 2A**). The Nuclear/Cytosolic (N/C) ratio was increased upon stretch. Our findings demonstrate that upon hypertrophic cyclic mechanical stretch, NFkB signaling is activated in ARCMs.

Cyclic mechanical stretch induces activation of both MAPK/ERK1/2 [27], [28] and PI3K [29] pathways in neonatal cardiomyocytes. In a similar manner in ARCMs, we found that hypertrophic cyclic stretch promoted an increase in the activation of the MAPK/ERK1/2 pathway as indicated by increased phospho-ERK1/2 (**Figure 2B**). Cyclic mechanical stretch also activated the PI3K pathway as demonstrated by an increase in phospho-AKT levels (**Figure 2C**). Taken together, we find that in adult cardiomyocytes cyclic mechanical stretch activates three primary pathways involved in the hypertrophic responses.

### The MAPK/ERK1/2 and PI3K pathways do not regulate stretch-mediated VEGF secretion

**Figure 3:**
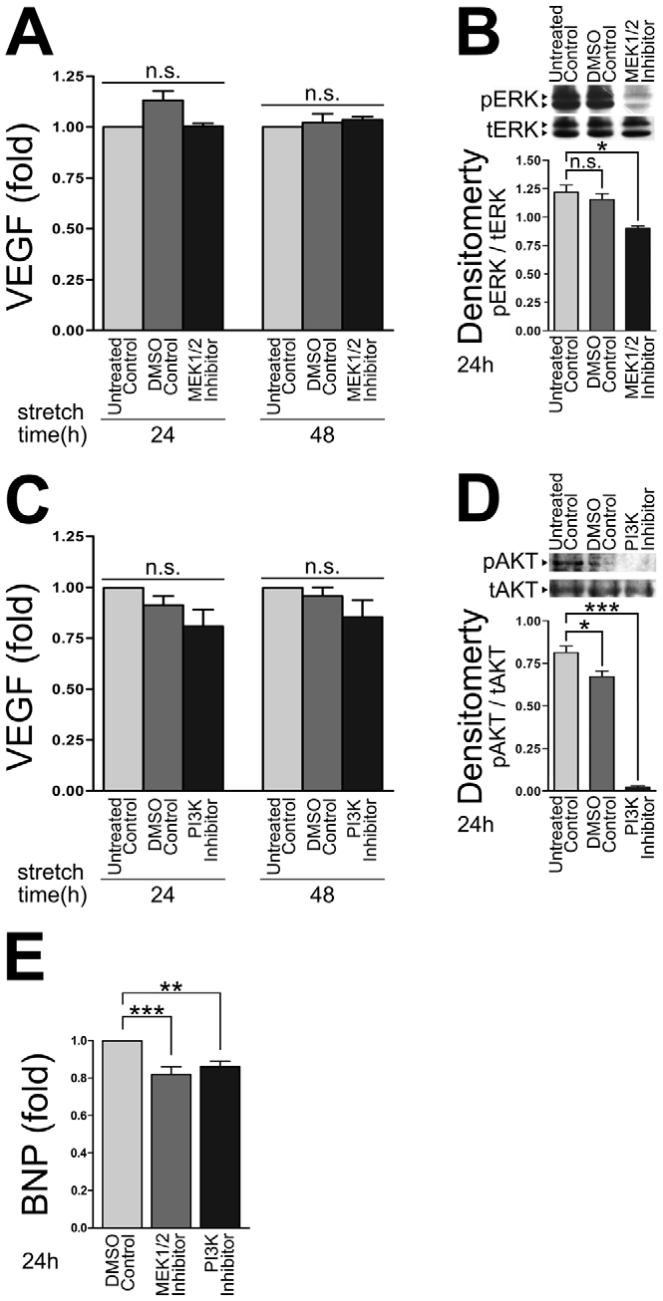
Cyclic mechanical stretch-induced VEGF secretion is independent of the MAPK/ERK1/2 or PI3K pathways. Inhibition of either the MAPK/ERK1/2 or PI3K signaling pathways in isolated ARCMs did not block VEGF secretion induced by cyclic mechanical stretch. Isolated ARCMs attached to laminin for 24 h were treated with the ERK1/2 kinase inhibitor (U0126; 0.5 µM) or DMSO-carrier control and subjected to (**A**) 24 h or 48 h of cyclic mechanical stretch (10% stretch) or no stretch (0% stretch). ELISA was used to determine the amount of VEGF secreted into the media. (**B**) The ERK1/2 kinase inhibitor was active in isolated primary ARCMs as pERK1/2 levels decreased in cells treated with the ERK1/2 kinase inhibitor (U0126; 0.5 µM) compared to the DMSO-carrier control. Stretch-activated VEGF secretion is independent of PI3K. ARCMs attached to laminin for 24 h were treated with the PI3K inhibitor (LY294002; 0.5 µM) or DMSO-carrier control and subjected to 24 h or 48 h (**C**) of cyclic mechanical stretch (10% stretch) or no stretch (0% stretch). ELISA was used to determine the amount of VEGF secreted into the media. (**D**) The PI3K inhibitor (LY294002; 0.5 µM) was active in isolated ARCMs as pAKT levels decreased in cells treated with the PI3K inhibitor (LY294002; 0.5 µM) compared to the DMSO-carrier control. Relative intensity of phospho-ERK1/2/total ERK1/2 (pERK/tERK) or phospho-AKT/total AKT (pAKT/tAKT) was determined. (**E**) ELISA was used to determine the amount of BNP secreted into the media. Treatment with the either ERK1/2 (U0126) or PI3K (LY294002) inhibitors blocked BNP secretion at 24 hours compared to DMSO-carrier control. ***P<0.001; One-Way ANOVA with a Bonferroni post-test was used to determine the statistical significance of data. n.s. = not significant. The values represent the average of three independent experiments.

Because both the MAPK/ERK1/2 pathway and the PI3K pathway regulate VEGF expression in a cell type specific manner [30], we next examined whether either pathway was involved in promoting stretch-induced VEGF secretion in ARCMs. Chemical inhibitors against each pathway were tested in our *in vitro* system. ARCMs bound to laminin were treated with either the ERK1/2 kinase inhibitor (U0126) or the PI3K inhibitor (LY294002), and subjected to 24 h or 48 h of cyclic mechanical stretch. Neither inhibition of ERK1/2 nor of PI3K abrogated the stretch-induced increase in VEGF secretion (**Figure 3A, C**). The ERK1/2 and PI3K inhibitors were active in primary ARCMs as treatment of these cells with either the ERK1/2 or PI3K inhibitor over 24 h significantly attenuated phospho-ERK1/2 (**Figure 3B**) and phospho-AKT levels (**Figure 3D**). To confirm that these inhibitors were active in these cells and that these pathways are important in hypertrophic signaling we also examined whether expression of BNP, a known marker of hypertrophy, was decreased upon inhibitor treatment. In agreement with published findings, the ERK1/2 and PI3K inhibitors significantly decreased BNP expression (**Figure 3E**). Taken together, these findings suggest that neither the MAPK/ERK1/2 nor the PI3K pathway is involved in cyclic mechanical stretch-induced secretion of VEGF in ARCMs but may be involved in the regulation of other hypertrophic signaling pathways.

### Inhibiting the NFkB pathway blocks mechanical stretch-induced VEGF secretion in adult cardiomyoyctes

**Figure 4:**
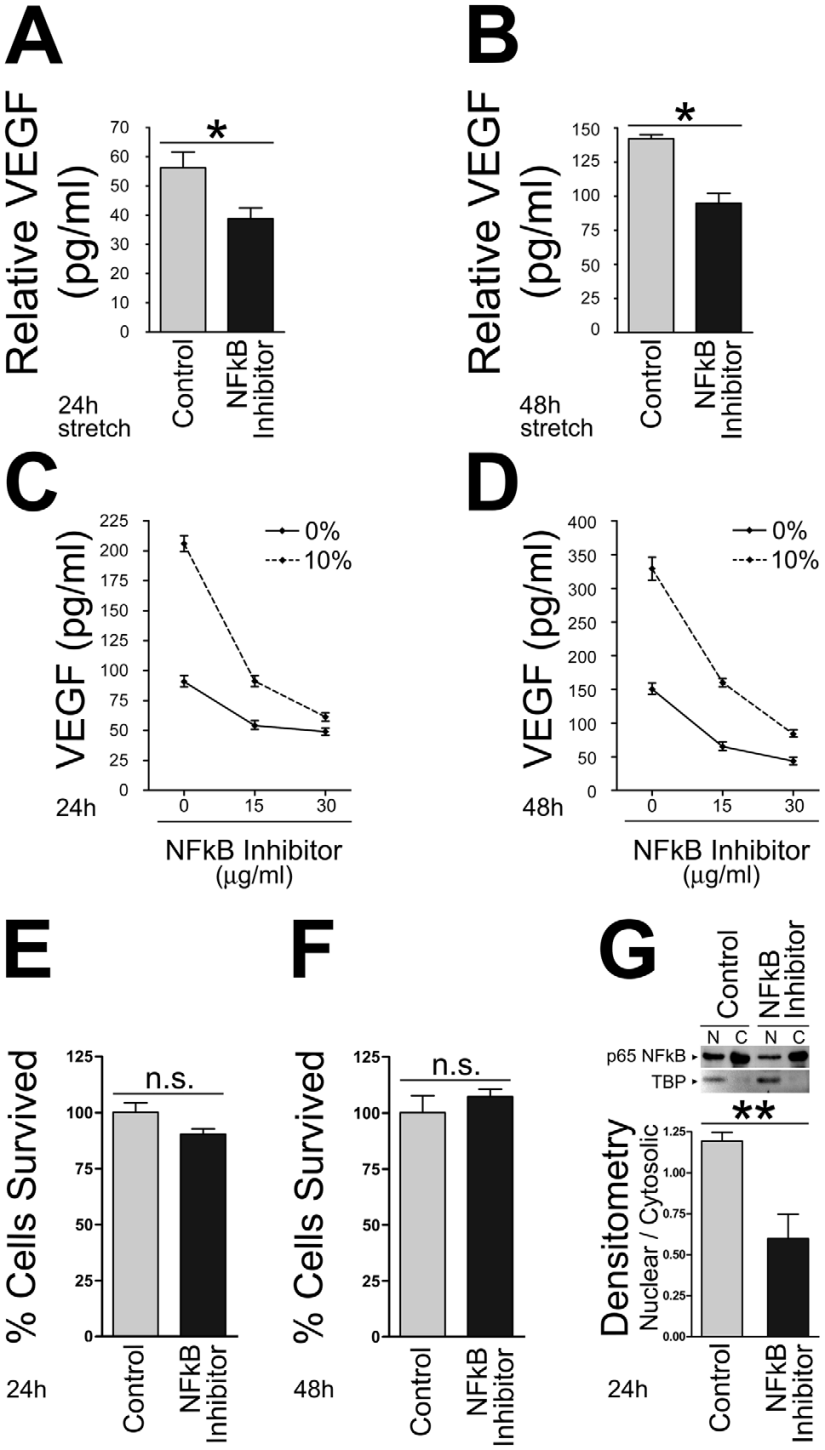
Inhibition of the NFkB pathway blocks cyclic mechanical stretch-induced VEGF secretion in a dose-dependent manner. Primary ARCMs cultured on laminin for 24 h were incubated with the NFkB inhibitor (SN50; 15 µg/ml) or its inactive control peptide (SN50M; 15 µg/ml), and subjected to 24 h or 48 h of cyclic mechanical stretch (10% stretch). Non-stretched control cells (0% stretch) were incubated under identical conditions. ELISA was used to analyze VEGF levels secreted into the culture media at 24 h (**A**) or 48 h (**B**). Inhibition of the NFkB pathway blocks cyclic mechanical stretch-induced VEGF secretion in a dose-dependent manner. Isolated primary ARCMs cultured on laminin were treated with NFkB inhibitor (SN50; 15 µg/ml or 30 µg/ml) and stretched (10% stretch) for 24 h (**C**) or 48 h (**D**). ELISA was used to analyze the concentration of VEGF in the media. Isolated primary ARCMs treated with the NFkB inhibitor (SN50) or its inactive peptide control (SN50M) remained viable at 24 h (**E**) and 48 h (**F**) of culture as assessed by the MTT cell viability assay. Fold induction in relative mitochondrial activity represents the number of viable cells present after each treatment. (**G**) The NFkB peptide inhibitor (SN50) was active in isolated ARCMS as p65 levels were reduced in the nuclear fraction upon inhibitor treatment compared to peptide control. ***P<0.001; One-Way ANOVA with a Bonferroni post-test was used to determine the statistical significance of data. n.s. = not significant. The values represent the average of three independent experiments.

VEGF secretion is dependent upon activation of the NFkB pathway in mesenchymal stem cells [31]. To investigate whether NFkB mediates stretch-induced secretion of VEGF in primary ARCMs, cells were treated with an NFkB peptide inhibitor or its inactive control peptide, and subjected to 24 h and 48 h of hypertrophic cyclic mechanical stretch (**Figure 4A–B**). Blocking the NFkB pathway resulted in a significant reduction of stretch-mediated VEGF secretion in ARCMs compared to non-stretched controls at 24 h and 48 h (**Figure 4A–B**). Furthermore, the NFkB inhibitor blocked stretch-mediated VEGF secretion in a dose dependent manner (**Figure 4C–D**). ARCMs treated with an NFkB inhibitor or its inactive control peptide remained viable after 24 h and 48 h in culture (**Figure 4E–F**) as determined by the MTT assay. The NFkB inhibitor was active in primary ARCMs as treatment of these cells with the NFkB peptide inhibitor significantly attenuated p65 nuclear translocation (**Figure 4G**).

**Figure 5:**
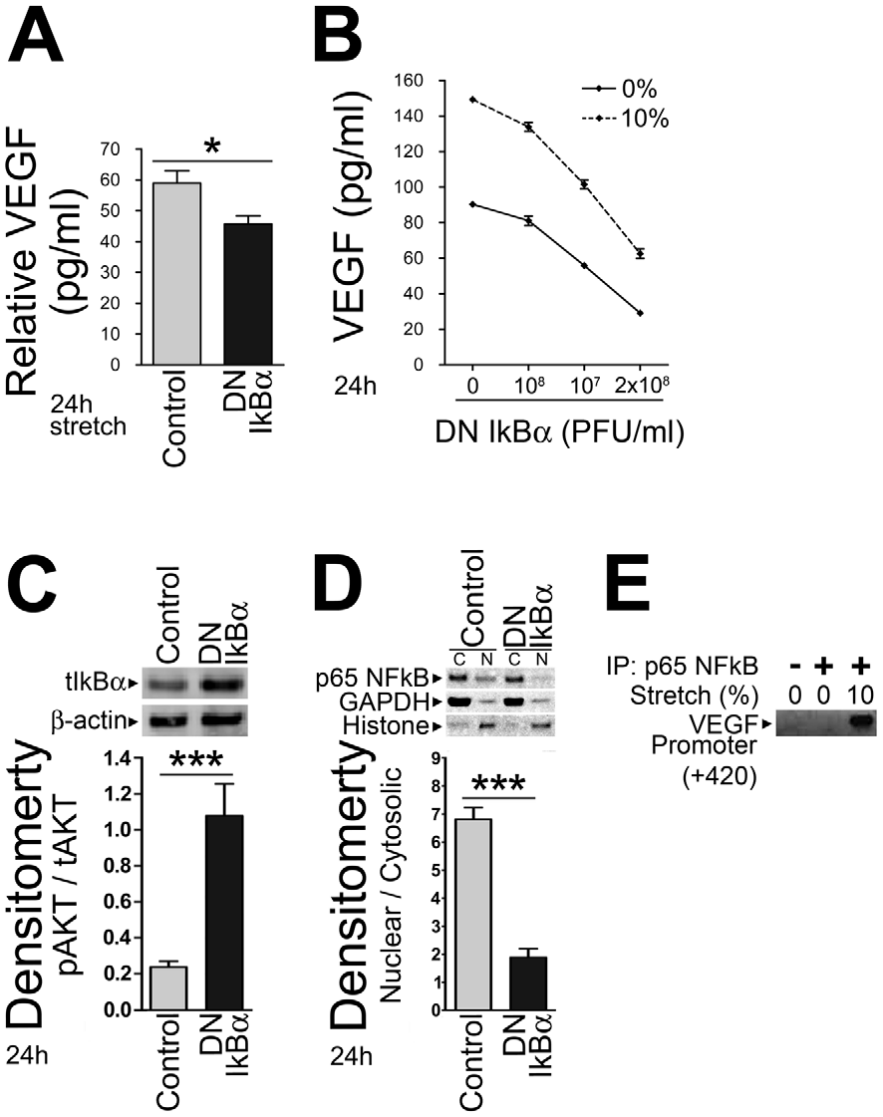
Expression of a dominant negative mutant IkBα blocks cyclic mechanical stretch-induced VEGF secretion in a dose-dependent manner. Primary ARCMs transduced with a recombinant adenovirus encoding an IkBα dominant negative mutant (DN IkBα) were cultured on laminin for 24 h and then subjected to 24 h of cyclic mechanical stretch (10% stretch). Non-stretched control cells (0% stretch) were incubated under identical conditions. ELISA was used to determine VEGF levels secreted into the culture media at 24 h (**A**). (**B**) Expression of the IkBα dominant negative mutant blocks cyclic mechanical stretch-induced VEGF secretion in a dose-dependent manner. ELISA was used to analyze the concentration of VEGF in the media. (**C**) The IkBα dominant negative mutant was expressed in isolated ARCMs as IkBα levels were significantly increased in ARCMs expressing the mutant compared to control ARCMs. (**D**) The IkBα dominant negative mutant was active in isolated ARCMs as p65 levels were significantly decreased in the nucleus of ARCMs expressing the mutant compared to control ARCMs. ***P<0.001; One-Way ANOVA with a Bonferroni post-test was used to determine the statistical significance of data. n.s. = not significant. The values represent the average of three independent experiments. (**E**) Representative ChIP assay PCR showing hypertrophic stretch increases NFkB binding to the native VEGF promoter in ARCMs. Protein-DNA complexes were immunoprecipitated with an NFkB p65 antibody followed by DNA isolation and purification and PCR. Non-immunoprecipitated chromatin was used as an “input” control (-). ARMCs subjected to 24 h stretch (10%) resulted in increased binding of NFkB to the VEGF promoter over 0% stretch.

We next used adenoviral gene transduction of a dominant negative mutant IkB to unequivocally demonstrate the involvement of NFkB signaling in the induction of stretch-mediated VEGF secretion. Twenty-four hours after isolation, ARCMs were transduced with a recombinant adenovirus encoding a dominant negative mutant form of IkBα or control and assayed for IkBα expression (**Figure 5C**). ARCMs expressing dominant negative IkBα were then subjected to 24 h of hypertrophic cyclic mechanical stretch. Inhibiting the NFkB pathway in this manner resulted in a significant reduction of stretch-mediated VEGF secretion in ARCMs compared to control at 24 h (**Figure 5A**). Furthermore, expression of dominant negative mutant IkB (DNM IkBα) blocked stretch-mediated VEGF secretion in a dose-dependent manner (**Figure 5B**). Infection of primary ARCMs with DNM IkBα significantly attenuated p65 nuclear translocation, indicating that it was active in these cells (**Figure 5D**). These findings support the hypothesis that stretch-induced VEGF secretion is mediated, at least in part, by the NFkB signal transduction pathway.

Finally, ChIP was used to determine whether hypertrophic stretch increases NFkB binding to the native VEGF promoter. Protein-DNA complexes were immunoprecipitated with an NFkB p65 antibody followed by DNA isolation, purification and PCR. Non-immunoprecipitated chromatin was used as an “input” control, and an IgG antibody control was performed on all occasions. PCR primers (VEGF promoter +420) were designed to cover only the fragment known to containing the two NFkB [32]. ARMCs subjected to 24 h stretch resulted in increased binding of NFkB to the VEGF promoter over IgG antibody control and 0% stretch (**Figure 5E**).

### VEGF expression is enhanced *in vivo* in the myocardium upon pressure overload induced hypertrophy

**Figure 6:**
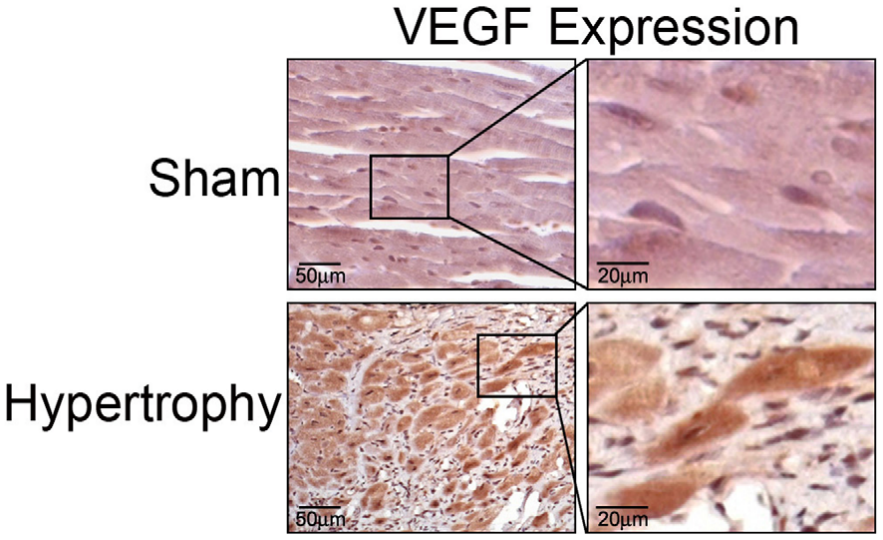
VEGF expression is enhanced in the myocardium and in cardiomyocytes upon pressure overload-induced hypertrophy in vivo. Immunostaining for VEGF using a polyclonal anti-VEGF antibody in paraffin fixed heart tissue from a pressure-overload hypertrophy model or sham control at day 10. Box =  higher magnification inset. Representative of three independent experiments.

Because the NFkB pathway is required for cardiomyocyte hypertrophic growth [33], we examined whether VEGF expression is enhanced *in vivo* in the myocardium upon induction of hypertrophy. Pressure overload-induced hypertrophy resulted in increased VEGF staining within the myocardium and in cardiomyocytes compared to Sham controls (**Figure 6**). This finding is in agreement with the literature that VEGF is involved in cardiomyocyte function and the hypertrophic response[13], [14], [15].

## Discussion

The present study demonstrates for the first time that hypertrophic mechanical stretch promotes VEGF secretion through the NFkB signal transduction pathway in adult rat cardiomyocytes. Blocking NFkB activation abrogates VEGF secretion induced by cyclic mechanical stretch in these cells. This is the first report to show a role for cyclic mechanical stretch in mediating VEGF secretion in adult cardiomyocytes. Moreover, this is the first demonstration that cyclic mechanical stretch promotes VEGF secretion through activation of the NFkB signaling pathway.

Stretch induced activation of NFkB is likely downstream of integrins because we see an increase in FAK activation in adult cardiomyocytes (data not shown) which is in agreement with previous findings in neonatal cardiomyocytes [16], [34]. Activation of the NFkB pathway is dependent upon the degradation of the inhibitory IkB protein and subsequent translocation of the NFkB complex to the nucleus where it activates gene transcription. We found that cyclic mechanical stretch promoted the nuclear translocation of NFkB and increased VEGF secretion in adult cardiomyocytes. Inhibition of NFkB signaling blocked stretch-mediated VEGF secretion. The NFkB pathway is involved in regulating the immediate early genes and is required for cardiomyocyte hypertrophic growth [33]. NFkB may promote cardiomyocyte growth by binding to the two NFkB recognition sites in the VEGF promoter [32]. Indeed, we demonstrate that in ARCMs subjected to stretch there is increased binding of NFkB to the VEGF promoter over non-stretch controls as determined by ChIP analysis. In our *in vitro* model system of stretch-induced hypertrophy [8], [9], [25] we found that NFkB activation is necessary for cyclic mechanical stretch-induced VEGF secretion. Hypoxia-induced stress also promotes NFkB-mediated VEGF secretion in human mesenchymal stem cells [31]. We now add that cyclic mechanical stretch promotes VEGF secretion via activation of the NFkB pathway in adult cardiomyocytes.

The MAPK/ERK1/2 and PI3K signal transduction pathways can also regulate VEGF expression, activate NFkB signaling and are activated in response to mechanical stretch in neonatal cardiomyocytes [27], [28], [29]. Our data indicate that in adult cardiomyocytes cyclic mechanical stretch activates both the MAPK/ERK1/2 and PI3K pathways. However, inhibiting these pathways did not block stretch-induced VEGF secretion. Primary ARCMs remained viable over 48 h with the addition of MAPK/ERK1/2 or PI3K inhibitors. These findings suggest that while both the MAPK/ERK1/2 and PI3K pathways may be involved in cyclic mechanical stretch mediated events such as BNP secretion, they are unlikely to be key modulators of stretch-induced VEGF secretion in adult cardiomyocytes. Interestingly, TGF-beta is secreted during stretch [12] and it has been previously reported that stretch-induced VEGF expression in rat neonatal cardiomyocytes is, at least in part, mediated by TGF-beta signaling [11], [35]. Here we show that stretch of adult rat cardiomyocytes promotes VEGF secretion via NFkB. TGF-beta activates the NFkB, MAPK/ERK1/2, and PI3K signaling pathways. Therefore, based on our data stretched-induced TGF-beta may activate VEGF secretion through its effects on NFkB. Taken together, our data suggest that the NFkB pathway plays a key role in regulating VEGF secretion in response to mechanical stretch.

VEGF is now considered a key modulator of cardiomyocyte function and the hypertrophic response. Both hypoxia and mechanical stress impact VEGF levels *in vivo* [13] and we found that pressure overload-induced hypertrophy increased VEGF expression in the myocardium *in vivo.* Taken together, our data indicate that cyclic mechanical stretch induces VEGF secretion in an NFkB-dependent manner in adult cardiomyocytes. Therefore, NFkB activation may play a central role in mediating VEGF secretion in response to hypertrophic stresses in the heart. Targeting this pathway may alleviate the pathological effects of hypertrophy and increase survival of patients presenting with hypertension or post-myocardial infarction.
